# Protein profile of well-differentiated versus un-differentiated human bronchial/tracheal epithelial cells

**DOI:** 10.1016/j.heliyon.2020.e04243

**Published:** 2020-06-23

**Authors:** Wen-Kuan Liu, Duo Xu, Yun Xu, Shu-Yan Qiu, Li Zhang, Hong-Kai Wu, Rong Zhou

**Affiliations:** State Key Laboratory of Respiratory Diseases, National Clinical Research Center for Respiratory Disease, The First Affiliated Hospital of Guangzhou Medical University, Guangzhou Institute of Respiratory Health, Guangzhou Medical University, Guangzhou, Guangdong, 510210, China

**Keywords:** Bioinformatics, Cell biology, Molecular biology, Human airway epithelium, Human primary bronchial/tracheal epithelial cell, Cell differentiation, Proteomics, Cell model

## Abstract

Un-differentiated (UD) and well-differentiated (WD) normal human primary bronchial/tracheal epithelial cells are important respiratory cell models. Mature, WD cells which can be derived by culturing UD cells at an air-liquid interface represent a good surrogate for *in vivo* human airway epithelium. The overall protein profile of WD cells is poorly understood; therefore, the current study evaluated the proteomic characteristics of WD and UD cells using label-free LC-MS/MS and LC-PRM/MS. A total of 3,579 proteins were identified in WD and UD cells. Of these, 198 proteins were identified as differentially expressed, with 121 proteins upregulated and 77 proteins downregulated in WD cells compared with UD cells. Differentially expressed proteins were mostly enriched in categories related to epithelial structure formation, cell cycle, and immunity. Fifteen KEGG pathways and protein interaction networks were enriched and identified. The current study provides a global protein profile of WD cells, and contributes to understanding the function of human airway epithelium.

## 1 Introduction

Human airway epithelium is a pseudostratified layer consisting of basal cells, secretory cells, and columnar ciliated cells. The epithelium provides a critical interface between the body and the external environment (Crystal et al., 2008). This epithelial layer is known to be necessary for host defense against inhaled particles and microbes. The layer serves as a physical barrier, secretes factors that mediate immunity, inflammation, and antioxidant defense, and clears materials through a mucociliary pathway (Diamond et al., 2000; Ghio et al., 2013; Kato and Schleimer, 2007; Ryu et al., 2010).

Generally, primary cell models are more representative of cells *in vivo*, compared with cancer-derived cell lines (Min et al., 2016; Thornton et al., 2000). Well-established organ-like primary cell models are more useful in investigating the functional properties of intact organs under normal or diseased conditions because these cells are likely to be more physiologically comparable to organs *in vivo* (Turner and Jones, 2009). In air-liquid interface (ALI) culture, un-differentiated normal human primary bronchial/tracheal epithelial (UD) cells can form a pseudostratified cell layer much like they do *in vivo* (Derichs et al., 2011). This well-differentiated normal human primary bronchial/tracheal epithelial (WD) cell model better mimics the *in vivo* environment than submersion culture which inhibits ciliogenesis and mucociliary movement (Min et al., 2016; Neugebauer et al., 2003). The WD cell model has been used for *in vitro* studies of drug pharmacokinetics and to study lung diseases such as asthma, chronic obstructive pulmonary disease and cystic fibrosis (Aghapour et al., 2018; Derichs et al., 2011; Gon and Hashimoto, 2018; Hackett et al., 2011; Hiemstra et al., 2018; Pickles, 2013; Schneider et al., 2010; Zhou et al., 2018). However, structural and proteomic differences between WD and UD cells remains to be characterized.

In the current study, we investigated the proteomic profiles of WD cells and UD cells using label-free Liquid Chromatography-Tandem Mass Spectrometry (LC-MS/MS). Our results can inform research on host pathogen infection and defense, external particle transport and clearance, and signal transduction.

## 2 Materials and methods

### 2.1 Cell culture

Normal human primary bronchial/tracheal epithelial cells which derived from an 8-year-old female with bacteria, yeast, fungi, *mycoplasma*, hepatitis B, hepatitis C and HIV testing negative were purchased from Lifeline (Passage #1, Lifeline, Frederick, MD, USA). Cells were cultured and passaged according to instructions provided by the supplier. Briefly, cells were thawed in a 37 °C water bath and cultivated in 75 cm^2^ flasks with serum-free growth media (BronchiaLife B/T complete medium, Lifeline, USA) at 37 °C, 5% CO_2_. Actively proliferating cells were passaged when at 70%–80% confluence. Passage #4 un-differentiated cells were divided into two parts, one was used for ALI culture, and the other was still used for submersion culture to obtain WD and UD cells samples for subsequent analysis, respectively.

Briefly, WD cells were grown at the ALI by seeding 5–8×10^4^ Passage #4 UD cells on collagen-coated transwell inserts (0.3 cm^2^, 0.4 μm pore size, BD-Falcon, Tewksbury, MA, USA) in 24-well plates at 37 °C, 5% CO_2_. Following 24 h of incubation, the medium in the apical chamber was removed by aspiration. Differentiation medium (Dulbecco's Modified Eagle Medium: Nutrient Mixture F-12 (DMEM:F12) containing 2% Ultroser G serum substitute (Pall BioSepra, Cergy-Staint-Christophe, France)) was added to the basolateral chamber as previously reported (Huang et al., 2012). Differentiation medium was replaced every 2 days, and WD cells were evaluated following 21 days of culture. UD cell samples were grown in submersion culture in three T75 flasks to 100% confluence prior to harvest for analysis.

### 2.2 Transepithelial electrical resistance (TEER) measurement

During culture of WD cells in differentiation medium, the polarity of cells was determined by TEER measurement. The apical and basolateral chambers of inserts were filled with fresh differentiation medium following 21 days of ALI culture, and equilibrated at 37 °C, 5% CO2 for 10 min. TEER values were determined using two Millicell-ERS (MERS00002, Millipore, Burlington, MA, USA) electrodes submerged into the insert medium. WD cells with TEER values > 1000 Ω cm^2^ were considered well-differentiated and met the requirement for subsequent studies of the model (Hiemstra et al., 2018; Huang et al., 2012; Min et al., 2016).

### 2.3 Immunofluorescence assay for WD cells biomarkers Zona occludens-1 and β-tubulin IV

To further confirm differentiation of WD cells, two differentiation biomarkers — tight junction protein Zona occludens-1 (ZO-1) and cilia marker β-tubulin IV — were quantified by immunofluorescence assay (IFA) (Huang et al., 2012). Following 21 days of ALI culture, WD cells on insert membranes were fixed with cold absolute ethanol for 20 min. Fixed membranes were cut into several small pieces, washed in PBS three times for 5 min, and permeabilized with 0.2% Triton X-100 for 15 min at room temperature. Membranes were blocked with 10% goat serum for 30 min at room temperature, then incubated overnight at 4 °C with primary ZO-1 (#13663, CST, Danvers, MA, USA) and β-tubulin IV (ab179509, Abcam, Cambridge, MA, USA) antibody diluted 1:200 and 1:400 in PBS plus 2% goat serum, respectively. Subsequently, membranes were incubated with a fluorescein Alexa fluor 488-conjugated secondary antibody (#4412, CST). Confocal images were captured using a D-Eclipse C1 confocal microscope (Nikon, Melville, NY, USA) controlled by Nikon EZ-C1 software.

### 2.4 Sample preparation for label-free LC-MS/MS experiments

Media was removed from UD and WD cells cultures, and cells were washed twice with HBSS. A total of 500 μl lysis buffer (4% SDS, 100 mM DTT, 150 mM Tris-HCl pH 8.0) was added to each T75 flask containing UD cells. Cells were then scraped and collected. UD cells from three T75 flasks were used for label-free LC-MS/MS experiments. Transwell inserts containing WD cells (0.3 cm^2^) with TEER values > 1000 Ω cm^2^ were collected. Eleven inserts of WD cells were torn off and placed together into 150 μl of lysis buffer. A total of 3 × 11 inserts of WD cells were used for label-free LC-MS/MS. Post-addition of lysis buffer, UD and WD cells were disrupted using a homogenizer (Fastprep-24®, MP Biomedical, Solon, OH, USA), then boiled for 5 min. Resulting homogenates were ultrasonicated and boiled again for 5 min. Undissolved cellular debris was removed by centrifugation at 14000 rpm for 15 min. The supernatant was collected and quantified with a BCA Protein Assay Kit (Bio-Rad, USA). Protein digestion (250 μg for each sample) was performed according to the FASP procedure described by Wisniewski, Zougman et al. (Wisniewski et al., 2009). Briefly, DTT and other low-molecular-weight components of the lysis buffer were removed by repeated centrifuge-facilitated ultrafiltration (Microcon units, 30 kD) using 200 μl of UA buffer (8 M Urea, 150 mM Tris-HCl, pH 8.0). Reduced cysteine residues were then blocked by incubating for 20 min with 100 μl of 0.05 M iodoacetamide in UA buffer in darkness. Filters were washed three times with 100 μl of UA buffer, then twice with 100 μl of 25 mM NH_4_HCO_3_. Finally, the protein suspension was digested overnight at 37 °C with 3 μg trypsin (Promega) in 40 μl of 25 mM NH_4_HCO_3_. Resulting peptides were collected as a filtrate and measured by UV light spectral density at 280 nm. Peptide content was calculated using an extinction coefficient on the basis of tryptophan and tyrosine frequency in vertebrate proteins.

### 2.5 Q exactive LC-MS/MS analysis

Peptide samples were desalted on C18 Cartridges (Empore™ SPE Cartridges C18 (standard density), bed I.D. 7 mm, volume 3 ml, Sigma), then concentrated by vacuum centrifugation and reconstituted in 40 μl of 0.1% (v/v) trifluoroacetic acid. MS experiments were performed on a Q Exactive mass spectrometer coupled to an Easy nLC (Proxeon Biosystems, now Thermo Fisher Scientific). Five μg of peptide were loaded onto a C18-reversed phase column (Thermo Scientific Easy Column, 10 cm long, 75 μm inner diameter, 3 μm resin) in buffer A (2% acetonitrile, 0.1% formic acid) and separated with a linear gradient of buffer B (80% acetonitrile, 0.1% formic acid). The flow rate was controlled by IntelliFlow technology at 250 nL/min over 120 min. MS data were acquired using a data-dependent top10 method dynamically choosing the most abundant precursor ions from the higher-energy collisional dissociation (HCD) fragmentation survey scan (300–1800 m/z). Target value was determined by predictive Automatic Gain Control (pAGC). Dynamic exclusion duration was 25 s. Survey scans were acquired at a resolution of 70,000 at m/z 200 and the resolution for HCD spectra was set to 17,500 at 200 m/z. Normalized collision energy was 30 eV and the underfill ratio, which specifies the minimum target value percentage likely to be reached at maximum fill time, was defined as 0.1%. The instrument was run with peptide recognition mode enabled.

### 2.6 Sequence database searching and data analysis

MS data were analyzed using MaxQuant software version 1.3.0.5. MS data were searched against the UniProtKB Homo Sapiens database (3,024,653 total entries, downloaded on 12/09/17). An initial search was set at a precursor mass window of 6 parts-per-million (ppm). The search followed an enzymatic cleavage rule of Trypsin/P and allowed for a maximum of two missed cleavage sites and a mass tolerance of 20 ppm for fragment ions. Carbamidomethylation of cysteine was defined as fixed modification, while protein N-terminal acetylation and methionine oxidation were defined as variable modifications for database searches. The cutoff global false discovery rate (FDR) for peptide and protein identification was set to 0.01. Label-free quantification was carried out in MaxQuant as previously described (Schwanhausser et al., 2011). Protein abundance was calculated based on normalized spectral protein intensity (LFQ intensity) (Luber et al., 2010).

### 2.7 Imputation of missing intensity values

Original quantitative protein intensities were converted to base 2 logarithms (log_2_). Missing values in the quantification were imputed by two methods. First, intensity values of the two groups were processed separately. For a protein with missing values in a group, if at least one sample had a quantitative value in the same group, the missing values were imputed using the K nearest neighbors (KNN) method (Troyanskaya et al., 2001). Proteins with missing values in all samples of one group remained. These missing values were imputed using the random tail imputation (RTI) method (Deeb et al., 2012) using Persues software set to “Replace missing values from normal distribution” (Tyanova et al., 2016) (Width = 0.3, Down shift = 1.8). The KNN method assumes that missing intensity values result from an unknown and complex combination of random processes and the values are imputed based on measured intensities in other samples from the same group. The RTI method assumes that low abundance proteins are close to the limit of detection of the instrument. Missing values are drawn from the tail of a truncated normal distribution, representative of proteins that are in low abundance (Lazar et al., 2016; Webb-Robertson et al., 2015).

### 2.8 Identification of up/down-regulated proteins

Log_2_ intensities, with imputed values, were converted to original numbers by multiplying by two. For each protein, the fold change ratio was computed by dividing average intensity of WD cells by average intensity of UD cells. Ratios and mean intensity values of all six samples were fed into Persues significance B analysis to identify significant outlier ratios (Cox and Mann, 2008). By computing FDR based on significance B, proteins with a Benjamini-Hochberg corrected p-value threshold of 0.05 were defined as up/down-regulated proteins.

### 2.9 Expression profile analysis

Gene ontology (GO) IDs and KEGG orthology (KO) IDs of proteins were obtained by querying the UniProtKB database (UniProt Consortium, 2018). The GO and KO IDs were used to classify proteins into categories (Ashburner et al., 2000; The Gene Ontology, 2017) and KEGG pathways (Kanehisa et al., 2017; Kanehisa and Goto, 2000), respectively. The number of proteins in each classification was counted. Fisher's and chi-square tests were used to assess significance. Categories and pathways with a greater percent of proteins up or down regulated relative to the full protein set and with a Fisher's p < 0.05, were considered significant. The enrichment factor (EF) was expressed as follow (1):

(1) EF = (Entry/Whole)_**DIFSet**_ / (Entry/Whole)_**EntireSet**_

Where Entry equals the number of proteins in a classification category, Whole equals the number of proteins in the entire functional classification system, DIFSet equals the up/down-regulated protein set and EntireSet equals the entire protein set. The UniProtKB protein accession number was used to query STRING (Szklarczyk et al., 2017) to identify interaction relationships between pairs of up/down-regulated proteins. Each protein was manually confirmed by a combination of protein name and protein description. The network of interaction relationships was illustrated by R package graph (Csardi and Nepusz, 2006).

### 2.10 Confirmation of differentially expressed proteins by liquid chromatography parallel reaction monitoring mass spectrometry (LC-PRM/MS)

To confirm the differentially expressed proteins identified by label-free analysis, the expression levels of selected proteins were further quantified by LC-PRM/MS analysis (Peterson et al., 2012). Briefly, UD and WD cell samples were collected and lysed as previously described. Peptides were prepared according to the label free protocol. Each sample was then spiked with an AQUA stable isotope peptide as an internal reference standard. Tryptic peptides were loaded on C18 stage tips for desalting prior to reversed-phase chromatography on an Easy nLC-1200 system (Thermo Scientific). LC gradients were run for 45 min with acetonitrile ranging from 5 to 35%. PRM analysis was performed on a Q Exactive Plus mass spectrometer (Thermo Scientific). Optimized collision energy, charge state, and retention times of the most significantly regulated peptides were generated experimentally using unique high intensity peptides and high confidence target proteins. The mass spectrometer was operated in positive ion mode, with the following parameters: The full MS1 scan was acquired with a resolution of 70000 (at 200 m/z), automatic gain control (ACG) target values of 3.0 × 10^−6^, and 250 ms maximum ion injection time. Full MS scans were followed by 20 PRM scans at 35000 resolution (at 200 m/z), AGC of 3.0 × 10^−6^ and 200 ms maximum injection time. Targeted peptides were isolated with a 2 THz window. Ion activation/dissociation was performed at normalized collision energy of 27 in an HCD collision cell. Raw data were analyzed using Skyline (MacCoss Lab, University of Washington) (MacLean et al., 2010). Signal intensities of individual peptide sequences for each significantly altered protein was quantified relative to each sample and normalized to a standard reference.

### 2.11 Statistical analysis

Significance B measure was used to identify up/down-regulated proteins in WD cells versus UD cells. T-tests were used to analyze LC-PRM/MS data, and confirm significant protein-expression differences between WD cells and UD cells. Fisher's and chi-square tests were used to detect the significance of enriched GO categories and KEGG pathways.

## 3 Results

### 3.1 Cell and sample preparation for label-free LC-MS/MS

**Figure 1:**
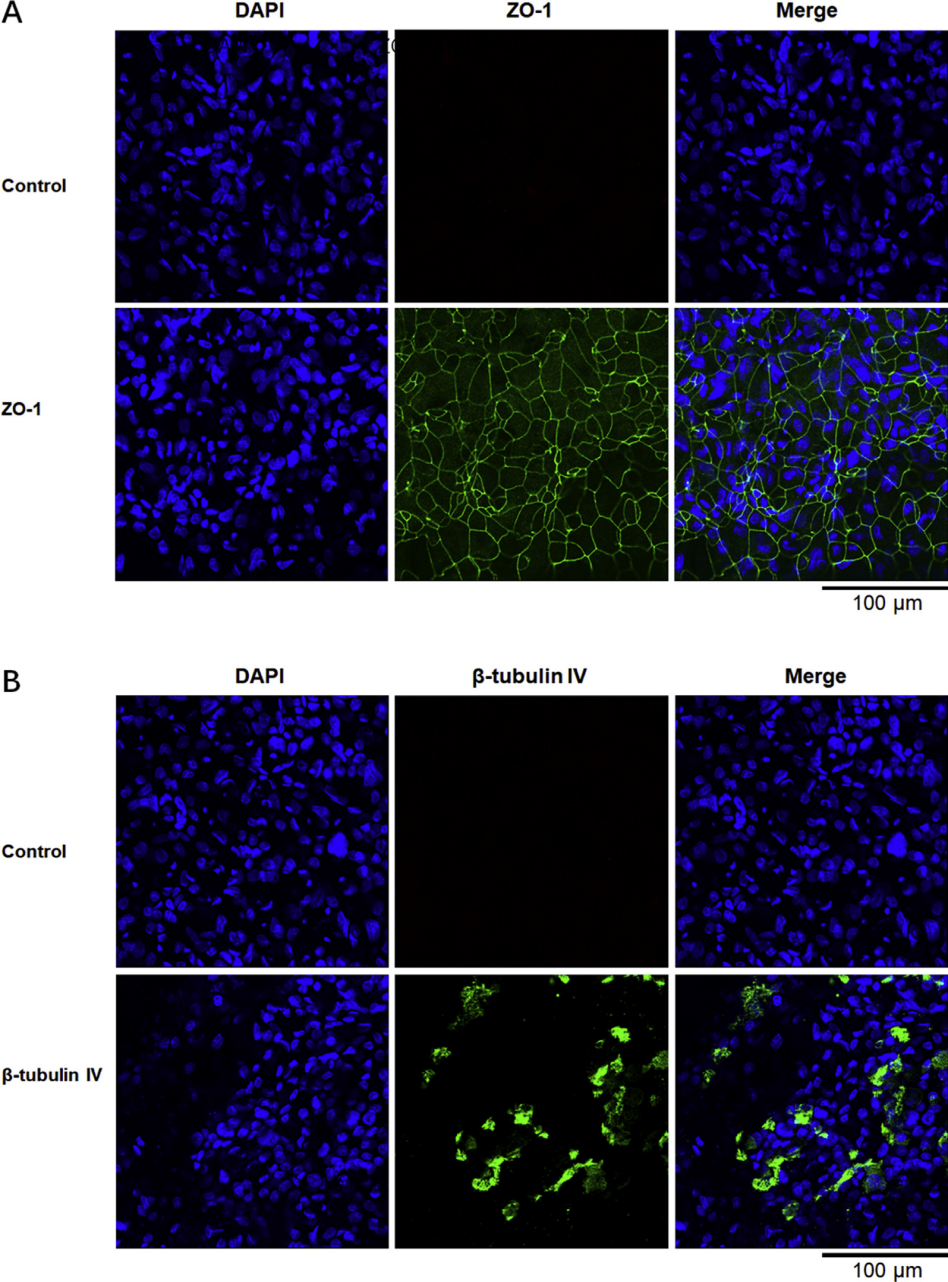
Immunofluorescence analysis of the tight junction marker ZO-1 and the cilia marker β-tubulin IV of well-differentiated normal human primary bronchial/tracheal epithelial cells. Well-differentiated normal human primary bronchial/tracheal epithelial cells were stained with anti-ZO-1 (A) or anti-β-tubulin IV antibody (B). Images were captured using a confocal microscope. Controls were stained with no primary antibody. Nuclei were stained with DAPI (blue).

A total of 33 WD cell inserts were cultured at ALI for 21 days. Mean TEER value was 1997 ± 454 Ω cm^2^, and each insert's TEER value was greater than 1000 Ω cm^2^. Expression of the biomarkers ZO-1 and β-tubulin IV was used to confirm differentiation to WD cells (Figure 1). Inserts were divided into 3 so that 11 inserts represented one sample. For UD cells, one T75 flask of cells represented one sample and three samples were evaluated. Lysis and extraction yielded a total of 337 ± 27 μg and 1385 ± 45 μg proteins from WD and UD cells, respectively. Proteins were digested and used for LC-MS/MS.

### 3.2 Data correlation, principal component analysis (PCA) and up/down-regulated proteins

**Figure S1:**
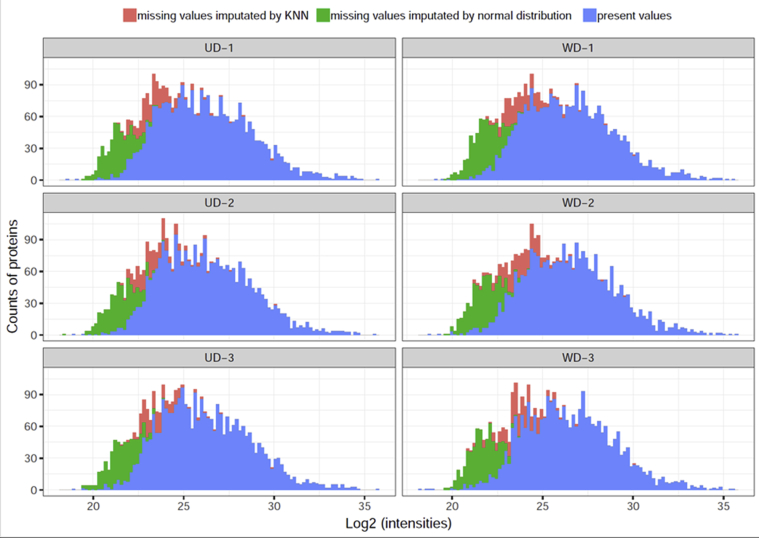
**Distribution of protein intensities in 6 samples of well-differentiated normal human primary bronchial/tracheal epithelial cells and un-differentiated cells**. UD and WD: un-differentiated and well-differentiated normal human primary bronchial/tracheal epithelial cells.

**Figure S2:**
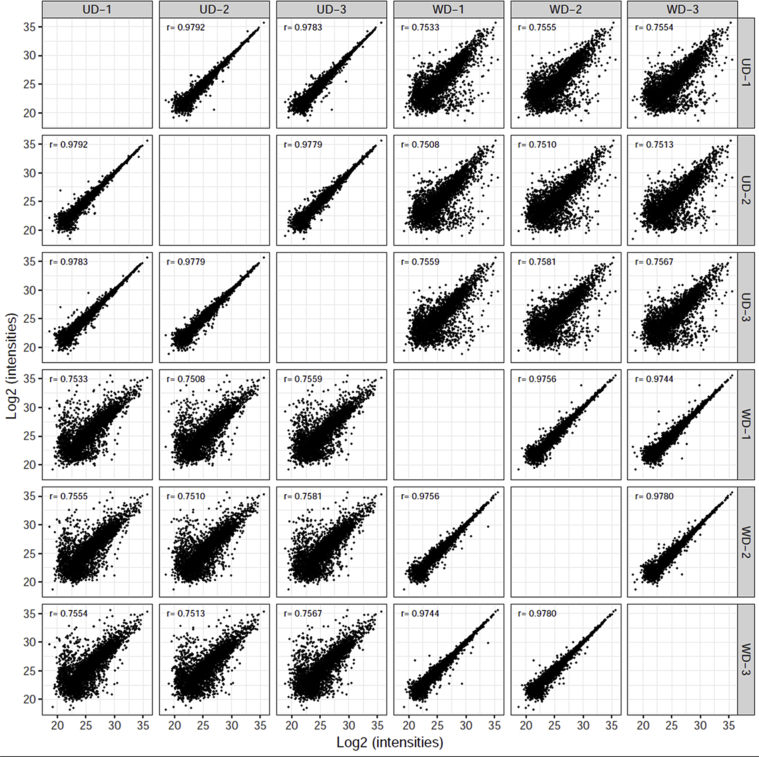
**Correlation plot and Pearson correlation coefficients of well-differentiated normal human primary bronchial/tracheal epithelial cells and un-differentiated cells binary comparisons**. UD and WD: un-differentiated and well-differentiated normal human primary bronchial/tracheal epithelial cells.

MS data analysis of the six samples identified 3,579 proteins after filtering out potential contaminating proteins. Log_2_ intensities, including imputed values, showed near normal distribution. Missing values imputed by the RTI method were distributed in areas of low intensity. These missing values existed in all samples within a group and were assumed to be due to low protein abundance (Figure S1). Pearson correlation coefficients were computed for every binary sample comparison. Within group correlation coefficients were greater than between group comparisons (r = 0.9744–0.9792 vs. r = 0.7508–0.7581) (Figure S2).

**Figure 2:**
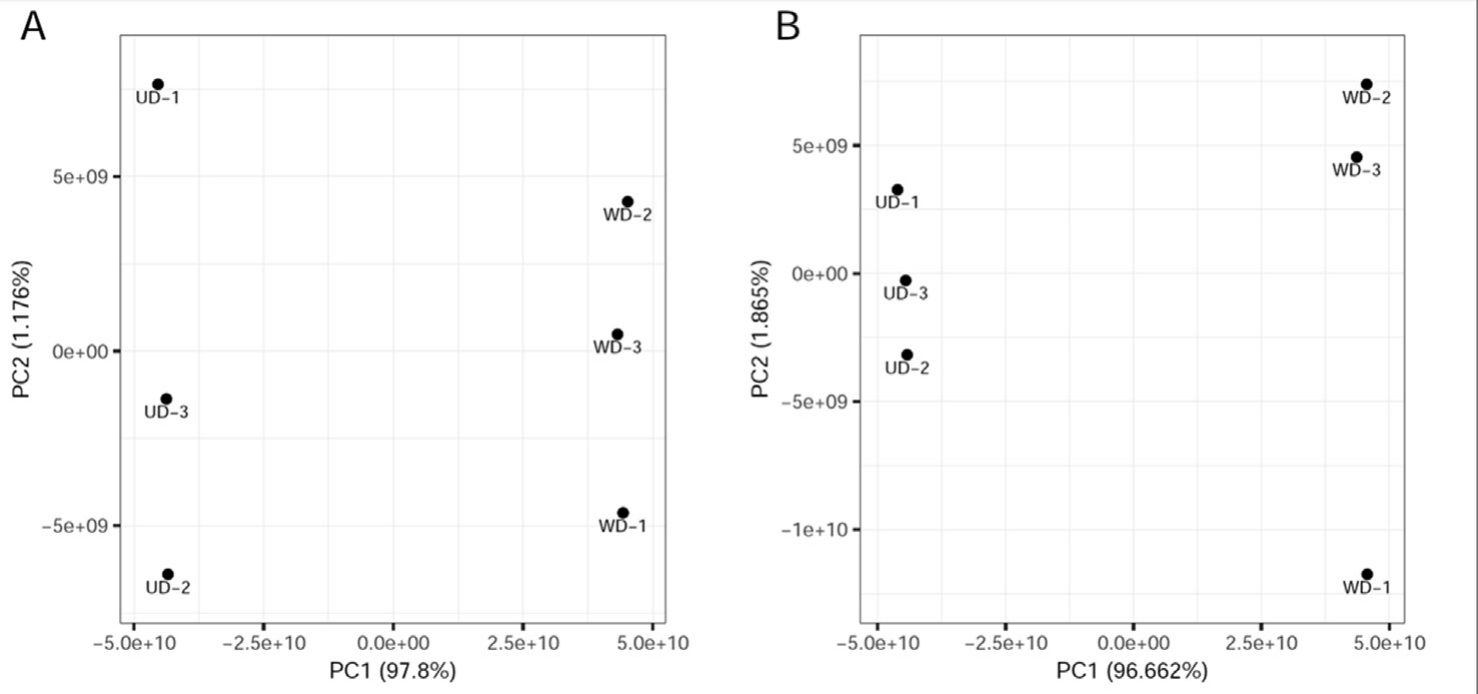
The first and second principal components derived from PCA of un-differentiated normal human primary bronchial/tracheal epithelial cells and well-differentiated cells. Percentages in parentheses represent the proportion of variances for PC1 or PC2. (A) Proteins with missing intensity values in any of the 6 samples were excluded. (B) All identified proteins were used after the imputation of missing values; UD and WD: un-differentiated and well-differentiated normal human primary bronchial/tracheal epithelial cells.

PCA was used to investigate the characteristics of abundant proteins identified in the 6 samples (Figure 2). Samples were plotted on a two-dimensional plane based on the coordinates obtained from the first and second principal components. Samples from the two cell types separated from each other along the x-axis (PCA1). This separation along the first principal component was observed regardless of whether the input data excluded missing values (Figure 2A) or consisted of all values, including imputed intensities (Figure 2B).

**Figure 3:**
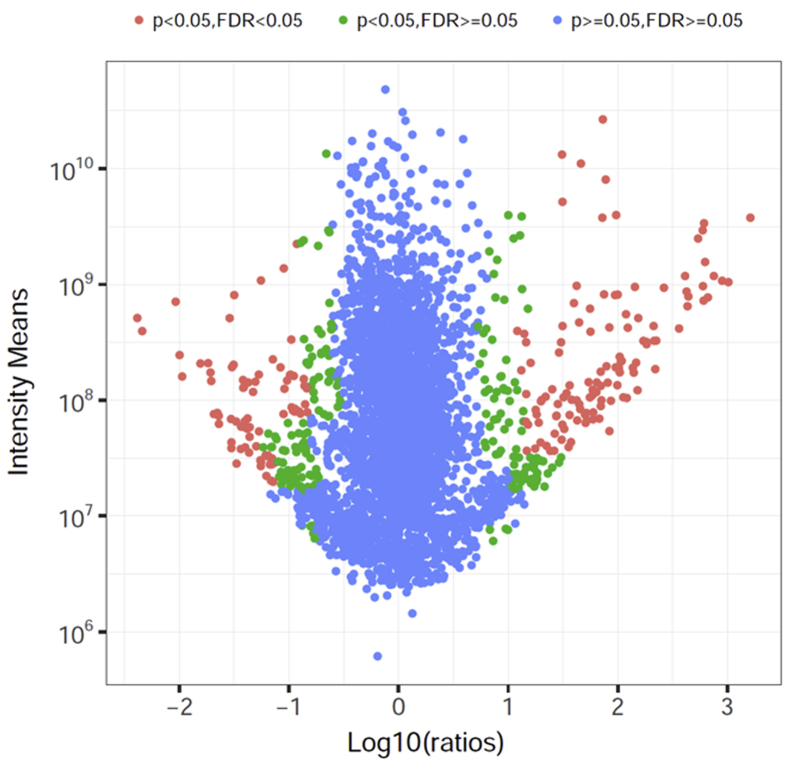
Proteome-wide quantification and significant fold change in well-differentiated normal human primary bronchial/tracheal epithelial cells compared with un-differentiated cells. Each dot represents a protein. The X-axis is the Log_10_ ratio obtained by dividing the mean of each protein's value in well-differentiated normal human primary bronchial/tracheal epithelial cells by its value in un-differentiated cells. FDR = false discovery rate.

The ratios of protein fold changes between the two groups were investigated by significance B measure (Figure 3). Proteins with significant fold changes appear in both high and low ratio regions. The boundaries between proteins of different significance ranges were not on a vertical line since significance B was weighted by signal intensity. Proteins with p < 0.05 and FDR<0.05 (red dots) were considered to be up/down-regulated proteins in this study. There were 198 such proteins, 121 (61.1%) of which were up-regulated (Table 1) and 77 (38.9%) down-regulated (Table 2) in WD cells compared with UD cells.

**Table 1:** Up-regulated proteins in well-differentiated normal human primary bronchial/tracheal epithelial cells compared with un-differentiated cells.

Serial number	UniProtKB accession	Protein name	Mean intensity	Log10 (Ratio)	p Value	FDR	Protein description
1	Q92817	EVPL	2.2630E+10	3.2090	8.7280E-14	1.0412E-10	Envoplakin
2	Q6UWP8	SBSN	6.2868E+09	3.0091	2.5710E-12	1.5336E-09	Suprabasin
3	O43251	RBFOX2	6.4577E+09	2.9513	6.5859E-12	2.9504E-09	RNA binding protein fox-1 homolog 2
4	Q9UBG3	CRNN	7.0967E+09	2.8757	2.1926E-11	8.7191E-09	Cornulin
5	P00966	ASS1	4.6387E+09	2.8216	5.0932E-11	1.8228E-08	Argininosuccinate synthase
6	A8K2U0	A2ML1	9.3797E+09	2.7958	7.5706E-11	2.2429E-08	Alpha-2-macroglobulin-like protein 1
7	P07476	IVL	2.0278E+10	2.7865	8.7285E-11	2.2429E-08	Involucrin
8	Q6ZVX7	NCCRP1	4.3506E+09	2.7824	9.2840E-11	2.2429E-08	F-box only protein 50
9	P01024	C3	5.8033E+09	2.7765	1.0152E-10	2.2429E-08	Complement C3
10	Q9UBC9	SPRR3	1.7624E+10	2.7743	1.0499E-10	2.2429E-08	Small proline-rich protein 3
11	P29508	SERPINB3	1.4966E+10	2.7322	1.9824E-10	3.3785E-08	Serpin B3
12	B2R853		4.7231E+09	2.6436	7.3169E-10	1.0475E-07	highly similar to Homo sapiens keratin 6E (KRT6E)
13	P15941	MUC1	3.8921E+09	2.6359	8.1770E-10	1.1091E-07	Mucin-1
14	O95171	SCEL	5.2113E+09	2.6272	9.2712E-10	1.1851E-07	Sciellin
15	P80188	LCN2	7.0981E+09	2.6134	1.1312E-09	1.3496E-07	Neutrophil gelatinase-associated lipocalin
16	P03973	SLPI	2.4978E+09	2.5587	2.1197E-12	1.5173E-09	Antileukoproteinase
17	P22735	TGM1	5.6235E+09	2.4204	1.6425E-08	1.4696E-06	Protein-glutamine gamma-glutamyltransferase K
18	O00748	CES2	1.9635E+09	2.3506	1.0653E-10	2.2429E-08	Cocaine esterase
19	Q96SN8	CDK5RAP2	1.1139E+09	2.3418	1.2474E-10	2.4803E-08	CDK5 regulatory subunit-associated protein 2
20	B7ZLF8		1.9555E+09	2.3314	1.5051E-10	2.8351E-08	Uncharacterized protein
21	P01833	PIGR	2.6257E+09	2.3264	1.6455E-10	2.9447E-08	Polymeric immunoglobulin receptor
22	Q9BYD5	CNFN	1.8270E+09	2.2626	5.0755E-10	8.0493E-08	Cornifelin
23	Q14CN2	CLCA4	1.9156E+09	2.2615	5.1728E-10	8.0493E-08	Calcium-activated chloride channel regulator 4
24	Q9UBD6	RHCG	1.9540E+09	2.2337	8.3673E-10	1.1091E-07	Ammonium transporter Rh type C
25	P32926	DSG3	3.0762E+09	2.1866	3.2491E-07	1.9710E-05	Desmoglein-3
26	P0C870	JMJD7	7.3191E+08	2.1801	8.4130E-09	8.1379E-07	JmjC domain-containing protein 7
27	Q8TE68	EPS8L1	1.2669E+09	2.1670	2.5945E-09	2.9018E-07	Epidermal growth factor receptor kinase substrate 8-like protein 1
28	P07099	EPHX1	5.7145E+09	2.1557	4.7195E-07	2.7244E-05	Epoxide hydrolase 1
29	Q9H8H3	METTL7A	1.0383E+09	2.1458	3.6872E-09	3.7705E-07	Methyltransferase-like protein 7A
30	P19957	PI3	1.1453E+09	2.1367	4.2885E-09	4.2635E-07	Elafin
31	P33121	ACSL1	2.5380E+09	2.0909	9.0582E-09	8.5314E-07	Long-chain-fatty-acid-CoA ligase 1
32	O00204	SULT2B1	6.2752E+08	2.0820	3.8512E-08	2.9327E-06	Sulfotransferase family cytosolic 2B member 1
33	P09758	TACSTD2	3.3186E+09	2.0749	1.2247E-06	6.1733E-05	Tumor-associated calcium signal transducer 2
34	C9JRL4	MDH1	6.3613E+08	2.0502	6.2117E-08	4.4464E-06	Malate dehydrogenase, cytoplasmic
35	O15020	SPTBN2	1.3119E+09	2.0354	2.1974E-08	1.8725E-06	Spectrin beta chain, non-erythrocytic 2
36	P00751	CFB	1.2925E+09	2.0267	2.5197E-08	2.0972E-06	Complement factor B
37	O43653	PSCA	1.0439E+09	2.0179	2.8935E-08	2.3205E-06	Prostate stem cell antigen
38	P40199	CEACAM6	1.4252E+09	2.0174	2.9176E-08	2.3205E-06	Carcinoembryonic antigen-related cell adhesion molecule 6
39	Q7L5L3	GDPD3	1.1362E+09	2.0105	3.2449E-08	2.5246E-06	Lysophospholipase D GDPD3
40	Q9NZT1	CALML5	4.9035E+09	1.9990	2.9067E-06	1.3168E-04	Calmodulin-like protein 5
41	Q9NQ38	SPINK5	8.0895E+08	1.9956	1.3895E-07	9.5636E-06	Serine protease inhibitor Kazal-type 5
42	Q8WWI1	LMO7	1.1466E+09	1.9920	4.3241E-08	3.2242E-06	LIM domain only protein 7
43	P35321	SPRR1A	2.3863E+10	1.9850	3.3994E-06	1.4658E-04	Cornifin-A
44	P02511	CRYAB	1.1546E+09	1.9809	5.1321E-08	3.7485E-06	Alpha-crystallin B chain
45	O60218	AKR1B10	4.8585E+09	1.9748	3.8097E-06	1.6232E-04	Aldo-keto reductase family 1 member B10
46	B3KVV6		5.9357E+08	1.9369	3.2259E-07	1.9710E-05	highly similar to Homo sapiens alpha-2-macroglobulin-like 1 (A2ML1)
47	P51178	PLCD1	3.2467E+08	1.9235	4.5173E-07	2.6504E-05	1-phosphatidylinositol 4,5-bisphosphate phosphodiesterase delta-1
48	P06731	CEACAM5	2.5495E+09	1.9228	1.2376E-07	8.6848E-06	Carcinoembryonic antigen-related cell adhesion molecule 5
49	Q13228	SELENBP1	8.5095E+08	1.9082	4.8299E-07	2.7439E-05	Methanethiol oxidase
50	Q14002	CEACAM7	1.1786E+09	1.8971	1.8142E-07	1.2251E-05	Carcinoembryonic antigen-related cell adhesion molecule 7
51	O60437	PPL	4.8356E+10	1.8896	9.6145E-06	3.7813E-04	Periplakin
52	Q13835	PKP1	4.9289E+09	1.8745	1.1282E-05	4.3888E-04	Plakophilin-1
53	P06702	S100A9	1.5995E+11	1.8634	1.2687E-05	4.8826E-04	Protein S100-A9
54	P30838	ALDH3A1	2.2648E+10	1.8587	1.3318E-05	5.0174E-04	Aldehyde dehydrogenase, dimeric NADP-preferring
55	P57735	RAB25	5.9955E+08	1.8507	1.0653E-06	5.6068E-05	Ras-related protein Rab-25
56	Q01995	TAGLN	7.9855E+08	1.8454	1.1443E-06	5.8876E-05	Transgelin
57	Q5K634		5.3396E+08	1.8449	1.1515E-06	5.8876E-05	SCCA2/SCCA1 fusion protein isoform 1
58	P12277	CKB	1.0580E+09	1.8435	3.9578E-07	2.3608E-05	Creatine kinase B-type
59	Q5K684		4.1629E+08	1.8313	1.5625E-06	7.5569E-05	SCCA1/SCCA2 fusion protein
60	T2F9S8	UPK3BL1	8.5360E+08	1.8080	1.8864E-06	9.0017E-05	Uroplakin-3b-like protein 1
61	O14493	CLDN4	7.0612E+08	1.8047	1.9711E-06	9.2825E-05	Claudin-4
62	Q08AI8	C2orf54	4.7127E+08	1.7755	2.8922E-06	1.3168E-04	Uncharacterized protein C2orf54
63	P11684	SCGB1A1	4.1025E+08	1.7738	3.2945E-06	1.4379E-04	Uteroglobin
64	O76027	ANXA9	7.3907E+08	1.7704	3.0905E-06	1.3826E-04	Annexin A9
65	Q14802	FXYD3	6.6276E+08	1.7691	3.1462E-06	1.3901E-04	FXYD domain-containing ion transport regulator 3
66	Q99102	MUC4	2.3456E+09	1.7506	1.4571E-06	7.1440E-05	Mucin-4
67	Q13510	ASAH1	3.7070E+09	1.7466	4.1852E-05	1.4131E-03	Acid ceramidase
68	P04233	CD74	6.3419E+08	1.7451	4.2869E-06	1.8050E-04	HLA class II histocompatibility antigen gamma chain
69	Q8WVV4	POF1B	8.6002E+08	1.7334	4.9758E-06	2.0707E-04	Protein POF1B
70	A0A1B0GVI3	KRT10	4.0145E+08	1.7181	6.6414E-06	2.7011E-04	Keratin, type I cytoskeletal 10
71	P00352	ALDH1A1	4.6622E+08	1.7131	6.4414E-06	2.6499E-04	Retinal dehydrogenase 1
72	O60879	DIAPH2	3.8248E+08	1.7058	7.7328E-06	3.0751E-04	Protein diaphanous homolog 2
73	Q9BPW9	DHRS9	4.5132E+08	1.7001	7.5849E-06	3.0501E-04	Dehydrogenase/reductase SDR family member 9
74	P05109	S100A8	6.6455E+10	1.6634	9.3945E-05	2.9237E-03	Protein S100-A8
75	Q969L2	MAL2	5.0317E+08	1.6571	1.2900E-05	4.9118E-04	Protein MAL2
76	Q8N3Y7	SDR16C5	5.5245E+08	1.6492	1.4202E-05	5.2946E-04	Epidermal retinol dehydrogenase 2
77	P24821	TNC	2.8193E+09	1.6472	1.0950E-04	3.3212E-03	Tenascin
78	Q99878	HIST1H2AJ	4.0203E+08	1.6299	1.9310E-05	7.0521E-04	Histone H2A type 1-J
79	Q562Z4	ACT	5.9844E+08	1.6251	1.9022E-05	7.0185E-04	Actin-like protein
80	P22532	SPRR2D	5.8401E+09	1.6246	1.3536E-04	4.0037E-03	Small proline-rich protein 2D
81	P22528	SPRR1B	4.1411E+09	1.5997	1.7035E-04	4.8774E-03	Cornifin-B
82	Q16762	TST	6.0534E+08	1.5794	3.2663E-05	1.1674E-03	Thiosulfate sulfurtransferase
83	Q16610	ECM1	2.6063E+08	1.5727	3.7547E-05	1.3133E-03	Extracellular matrix protein 1
84	B3KUB6		8.0577E+08	1.5603	4.0782E-05	1.3953E-03	highly similar to Band 4.1-like protein 1
85	P09668	CTSH	4.4739E+08	1.5600	4.0936E-05	1.3953E-03	Pro-cathepsin H
86	P57730	CARD18	2.3624E+08	1.5511	4.8027E-05	1.5916E-03	Caspase recruitment domain-containing protein 18
87	P04066	FUCA1	6.8916E+08	1.5324	5.6178E-05	1.8278E-03	Tissue alpha-L-fucosidase
88	O15195	VILL	3.3877E+08	1.5111	7.5024E-05	2.4006E-03	Villin-like protein
89	O76041	NEBL	6.3585E+08	1.5051	7.6373E-05	2.4189E-03	Nebulette
90	Q9UIV8	SERPINB13	2.6246E+09	1.4959	3.7797E-05	1.3133E-03	Serpin B13
91	P31151	S100A7	3.1030E+10	1.4952	4.3317E-04	1.1194E-02	Protein S100-A7
92	Q5T2T1	MPP7	3.6873E+08	1.4946	8.9940E-05	2.8237E-03	MAGUK p55 subfamily member 7
93	P04792	HSPB1	7.9479E+10	1.4909	4.4937E-04	1.1488E-02	Heat shock protein beta-1
94	P04259	KRT6B	2.7359E+08	1.4878	9.6874E-05	2.9889E-03	Keratin, type II cytoskeletal 6B
95	P18510	IL1RN	1.8987E+09	1.4822	4.4462E-05	1.4872E-03	Interleukin-1 receptor antagonist protein
96	B4DRX0		1.5497E+09	1.4639	5.5077E-05	1.8085E-03	highly similar to guanylate binding protein family, member 6 (GBP6)
97	Q6UXB2	CXCL17	5.5635E+08	1.4538	1.3440E-04	4.0037E-03	C-X-C motif chemokine 17
98	Q92747	ARPC1A	4.4174E+08	1.4413	1.5382E-04	4.4758E-03	Actin-related protein 2/3 complex subunit 1A
99	Q9UN76	SLC6A14	4.3694E+08	1.4387	1.5821E-04	4.5664E-03	Sodium- and chloride-dependent neutral and basic amino acid transporter B(0+)
100	Q2I377		3.0828E+08	1.4248	1.8970E-04	5.3883E-03	Small proline rich protein
101	Q5Y7A7	HLA-DRB1	2.1967E+08	1.4198	1.9983E-04	5.5873E-03	HLA class II histocompatibility antigen, DRB1-13 beta chain
102	Q9C002	NMES1	7.4517E+08	1.4017	2.3416E-04	6.4465E-03	Normal mucosa of esophagus-specific gene 1 protein
103	O43240	KLK10	2.1896E+08	1.3837	2.8976E-04	7.9164E-03	Kallikrein-10
104	Q9UKR0	KLK12	2.4196E+08	1.3500	4.0703E-04	1.0712E-02	Kallikrein-12
105	P26447	S100A4	6.4331E+08	1.3415	4.3475E-04	1.1194E-02	Protein S100-A4
106	P40394	ADH7	6.0307E+08	1.3057	6.2060E-04	1.5213E-02	Alcohol dehydrogenase class 4 mu/sigma chain
107	O75841	UPK1B	5.8669E+08	1.2989	6.6380E-04	1.6161E-02	Uroplakin-1b
108	Q13938	CAPS	2.5889E+08	1.2952	6.9632E-04	1.6614E-02	Calcyphosin
109	Q8N335	GPD1L	3.8423E+08	1.2789	8.1387E-04	1.8915E-02	Glycerol-3-phosphate dehydrogenase 1-like protein
110	O60547	GMDS	4.5212E+08	1.2600	9.6618E-04	2.1612E-02	GDP-mannose 4,6 dehydratase
111	P10253	GAA	4.9534E+08	1.2507	1.0556E-03	2.3322E-02	Lysosomal alpha-glucosidase
112	P27338	MAOB	2.2924E+08	1.2461	1.1069E-03	2.4156E-02	Amine oxidase [flavin-containing] B
113	P00167	CYB5A	1.2616E+09	1.2046	8.8749E-04	2.0361E-02	Cytochrome b5
114	Q6ZNJ1	NBEAL2	3.6960E+08	1.1808	2.0022E-03	3.8320E-02	Neurobeachin-like protein 2
115	P12074	COX6A1	2.1947E+08	1.1694	2.2153E-03	4.1295E-02	Cytochrome c oxidase subunit 6A1, mitochondrial
116	Q15067	ACOX1	6.7813E+08	1.1645	2.3280E-03	4.2947E-02	Peroxisomal acyl-coenzyme A oxidase 1
117	O95833	CLIC3	1.8996E+09	1.1635	1.3201E-03	2.7559E-02	Chloride intracellular channel protein 3
118	Q3ZCW2	LGALSL	3.9168E+08	1.1614	2.3755E-03	4.3407E-02	Galectin-related protein
119	Q9UN36	NDRG2	2.2476E+09	1.1466	1.5480E-03	3.1125E-02	Protein NDRG2
120	P05120	SERPINB2	1.0883E+09	1.1198	1.9863E-03	3.8221E-02	Plasminogen activator inhibitor 2
121	P19971	TYMP	2.3800E+09	1.0834	2.7648E-03	4.9975E-02	Thymidine phosphorylase

FDR: false discovery rate.

**Table 2:** Down-regulated proteins in well-differentiated normal human primary bronchial/tracheal epithelial cells compared with un-differentiated cells.

Serial number	UniProtKB Accession	Protein Name	Mean Intensity	Log10 (Ratio)	p Value	FDR	Protein Description
1	P37268	FDFT1	3.0783E+09	-2.3824	7.1064E-16	2.5434E-12	Squalene synthase
2	Q9Y617	PSAT1	2.3742E+09	-2.3376	2.0480E-14	3.6650E-11	Phosphoserine aminotransferase
3	Q01581	HMGCS1	4.2580E+09	-2.0327	6.5950E-12	2.9504E-09	Hydroxymethylglutaryl-CoA synthase, cytoplasmic
4	P0DJJ0	SRGAP2C	1.4731E+09	-1.9967	6.7214E-11	2.1869E-08	SLIT-ROBO Rho GTPase-activating protein 2C
5	Q13642	FHL1	9.6190E+08	-1.9735	1.0290E-12	9.2067E-10	Four and a half LIM domains protein 1
6	A0A024R7D5	LDLR	1.2463E+09	-1.8067	3.5816E-09	3.7701E-07	Low density lipoprotein receptor (Familial hypercholesterolemia), isoform CRA_b
7	Q13509	TUBB3	1.2541E+09	-1.7364	1.4161E-08	1.2995E-06	Tubulin beta-3 chain
8	Q9NR30	DDX21	1.0413E+09	-1.7151	2.1298E-08	1.8591E-06	Nucleolar RNA helicase 2
9	O00622	CYR61	8.7896E+08	-1.7082	6.3728E-10	9.5035E-08	Protein CYR61
10	Q9NRN7	AASDHPPT	4.5529E+08	-1.6836	1.1057E-09	1.3496E-07	L-aminoadipate-semialdehyde dehydrogenase-phosphopantetheinyl transferase
11	Q8WWM9	CYGB	4.6634E+08	-1.6525	2.1998E-09	2.5397E-07	Cytoglobin
12	O95864	FADS2	4.4000E+08	-1.6397	2.9108E-09	3.1569E-07	Fatty acid desaturase 2
13	Q12805	EFEMP1	3.7486E+08	-1.6379	1.0847E-04	3.3180E-03	EGF-containing fibulin-like extracellular matrix protein 1
14	O43175	PHGDH	3.0724E+09	-1.5400	2.3106E-07	1.5314E-05	D-3-phosphoglycerate dehydrogenase
15	P46821	MAP1B	4.1297E+08	-1.5276	3.0452E-04	8.2566E-03	Microtubule-associated protein 1B
16	P33993	MCM7	2.3134E+08	-1.5244	3.1372E-04	8.4108E-03	DNA replication licensing factor MCM7
17	Q9Y2S6	TMA7	2.5980E+08	-1.5239	3.1490E-04	8.4108E-03	Translation machinery-associated protein 7
18	P49023	PXN	1.1578E+09	-1.5191	7.1610E-07	3.8832E-05	Paxillin
19	Q9UMD9	COL17A1	1.2158E+09	-1.5041	9.2116E-07	4.9207E-05	Collagen alpha-1(XVII) chain
20	P08133	ANXA6	4.8577E+09	-1.4983	4.9271E-07	2.7553E-05	Annexin A6
21	Q14683	SMC1A	3.9266E+08	-1.4805	4.6415E-04	1.1698E-02	Structural maintenance of chromosomes protein 1A
22	Q6NYC8	PPP1R18	1.7006E+08	-1.4773	7.5124E-05	2.4006E-03	Phostensin
23	P08243	ASNS	2.2748E+08	-1.4385	6.6961E-04	1.6193E-02	Asparagine synthetase [glutamine-hydrolyzing]
24	P46087	NOP2	3.5465E+08	-1.4361	6.8331E-04	1.6413E-02	Probable 28S rRNA (cytosine(4447)-C(5))-methyltransferase
25	Q03001	DST	8.9712E+08	-1.4183	2.6397E-07	1.7177E-05	Dystonin
26	P21589	NT5E	7.6840E+08	-1.4167	2.7252E-07	1.7417E-05	5′-nucleotidase
27	O60701	UGDH	7.7732E+08	-1.4106	3.0586E-07	1.9204E-05	UDP-glucose 6-dehydrogenase
28	P01583	IL1A	3.9459E+08	-1.4020	9.1373E-04	2.0830E-02	Interleukin-1 alpha
29	P50281	MMP14	3.4994E+08	-1.3994	9.3387E-04	2.1154E-02	Matrix metalloproteinase-14
30	P14317	HCLS1	2.3506E+08	-1.3983	9.4278E-04	2.1221E-02	Hematopoietic lineage cell-specific protein
31	O00148	DDX39A	3.4044E+08	-1.3797	1.1010E-03	2.4156E-02	ATP-dependent RNA helicase DDX39A
32	Q86V48	LUZP1	4.1773E+08	-1.3765	1.1304E-03	2.4371E-02	Leucine zipper protein 1
33	Q96QD8	SLC38A2	8.5362E+08	-1.3706	6.4406E-07	3.5463E-05	Sodium-coupled neutral amino acid transporter 2
34	P49736	MCM2	2.1169E+08	-1.3629	1.2641E-03	2.6931E-02	DNA replication licensing factor MCM2
35	Q8IVT2	MISP	2.8957E+08	-1.3581	1.3154E-03	2.7559E-02	Mitotic interactor and substrate of PLK1
36	Q14566	MCM6	3.5072E+08	-1.3573	1.3244E-03	2.7559E-02	DNA replication licensing factor MCM6
37	Q27J81	INF2	7.0837E+08	-1.3266	1.4321E-06	7.1190E-05	Inverted formin-2
38	Q9NX58	LYAR	8.6688E+08	-1.3062	2.0585E-06	9.5682E-05	Cell growth-regulating nucleolar protein
39	Q9H2H9	SLC38A1	2.3999E+08	-1.2974	2.1423E-03	4.0355E-02	Sodium-coupled neutral amino acid transporter 1
40	P17812	CTPS1	9.9882E+08	-1.2742	3.2945E-05	1.1674E-03	CTP synthase 1
41	Q04941	PLP2	3.2283E+08	-1.2726	2.5998E-03	4.7231E-02	Proteolipid protein 2
42	P84157	MXRA7	1.8172E+08	-1.2604	7.1016E-04	1.6832E-02	Matrix-remodeling-associated protein 7
43	O60232	SSSCA1	1.6184E+08	-1.2560	7.4088E-04	1.7445E-02	Sjoegren syndrome/scleroderma autoantigen 1
44	A0A0A6YYF2	LAMA3	6.4962E+09	-1.2550	2.7593E-05	9.9754E-04	HCG1811249, isoform CRA_e
45	Q92888	ARHGEF1	1.7622E+08	-1.2528	7.6439E-04	1.7881E-02	Rho guanine nucleotide exchange factor 1
46	P31146	CORO1A	2.0013E+08	-1.2128	1.1158E-03	2.4202E-02	Coronin-1A
47	P07942	LAMB1	1.3318E+08	-1.2053	1.1959E-03	2.5629E-02	Laminin subunit beta-1
48	P35080	PFN2	2.0038E+08	-1.1924	1.3479E-03	2.7885E-02	Profilin-2
49	Q9Y6A4	CFAP20	1.9573E+08	-1.1879	1.4044E-03	2.8723E-02	Cilia- and flagella-associated protein 20
50	O00461	GOLIM4	1.6700E+08	-1.1730	1.6080E-03	3.2150E-02	Golgi integral membrane protein 4
51	Q9NPR2	SEMA4B	1.2144E+08	-1.1639	1.7472E-03	3.4548E-02	Semaphorin-4B
52	Q14554	PDIA5	1.6747E+08	-1.1546	1.8978E-03	3.7015E-02	Protein disulfide-isomerase A5
53	P33992	MCM5	1.8888E+08	-1.1538	1.9124E-03	3.7015E-02	DNA replication licensing factor MCM5
54	B4DY32		1.3526E+09	-1.1459	1.9130E-04	5.3911E-03	highly similar to Asparagine synthetase (glutamine-hydrolyzing)
55	Q96CT7	CCDC124	1.1834E+08	-1.1437	2.0924E-03	3.9833E-02	Coiled-coil domain-containing protein 124
56	Q96SB4	SRPK1	1.7336E+08	-1.1292	2.3771E-03	4.3407E-02	SRSF protein kinase 1
57	Q13085	ACACA	1.1539E+09	-1.0766	4.6078E-04	1.1696E-02	Acetyl-CoA carboxylase 1
58	P39748	FEN1	4.5603E+08	-1.0488	1.2811E-04	3.8530E-03	Flap endonuclease 1
59	P06454	PTMA	8.2681E+09	-1.0466	5.0925E-04	1.2745E-02	Prothymosin alpha
60	P05114	HMGN1	7.5290E+08	-1.0404	1.4455E-04	4.2406E-03	Non-histone chromosomal protein HMG-14
61	Q96CX2	KCTD12	8.8738E+08	-1.0162	2.0413E-04	5.6633E-03	BTB/POZ domain-containing protein KCTD12
62	A0A024R3T8	PARP1	9.8939E+08	-0.9899	1.2938E-03	2.7400E-02	Poly [ADP-ribose] polymerase
63	Q16222	UAP1	9.9843E+08	-0.9849	1.3697E-03	2.8173E-02	UDP-N-acetylhexosamine pyrophosphorylase
64	P17301	ITGA2	2.0048E+09	-0.9778	1.4842E-03	3.0181E-02	Integrin alpha-2
65	O43592	XPOT	5.2138E+08	-0.9751	3.6079E-04	9.5648E-03	Exportin-T
66	P0DME0	SETSIP	4.8935E+08	-0.9631	4.2423E-04	1.1083E-02	Protein SETSIP
67	Q9UHD1	CHORDC1	9.7224E+08	-0.9552	1.9133E-03	3.7015E-02	Cysteine and histidine-rich domain-containing protein 1
68	Q01628	IFITM3	4.7824E+08	-0.9473	5.2393E-04	1.3022E-02	Interferon-induced transmembrane protein 3
69	Q9BQL6	FERMT1	5.0585E+08	-0.9445	5.4389E-04	1.3425E-02	Fermitin family homolog 1
70	Q13751	LAMB3	1.3431E+10	-0.9277	2.1612E-03	4.0498E-02	Laminin subunit beta-3
71	P49321	NASP	4.8148E+08	-0.9087	8.6662E-04	2.0010E-02	Nuclear autoantigenic sperm protein
72	Q15654	TRIP6	4.6028E+08	-0.8940	1.0450E-03	2.3231E-02	Thyroid receptor-interacting protein 6
73	P13726	F3	9.1561E+08	-0.8641	1.5165E-03	3.0665E-02	Tissue factor
74	P16949	STMN1	8.0051E+08	-0.8574	1.6455E-03	3.2717E-02	Stathmin
75	Q8WX93	PALLD	5.5338E+08	-0.8518	1.7609E-03	3.4628E-02	Palladin
76	P27708	CAD	7.5907E+08	-0.8364	2.1201E-03	4.0147E-02	CAD protein
77	B3KS36		4.7213E+08	-0.8319	2.2366E-03	4.1475E-02	highly similar toribosomal protein L3 (RPL3), transcript variant 2

FDR: false discovery rate.

### 3.3 Comparative proteomic analysis

Identified proteins were sorted using the gene function classification systems GO and KEGG pathways. The number of functional entries in the up/down-regulated protein set were then compared with the functional entries in the entire protein set.

**Figure 4:**
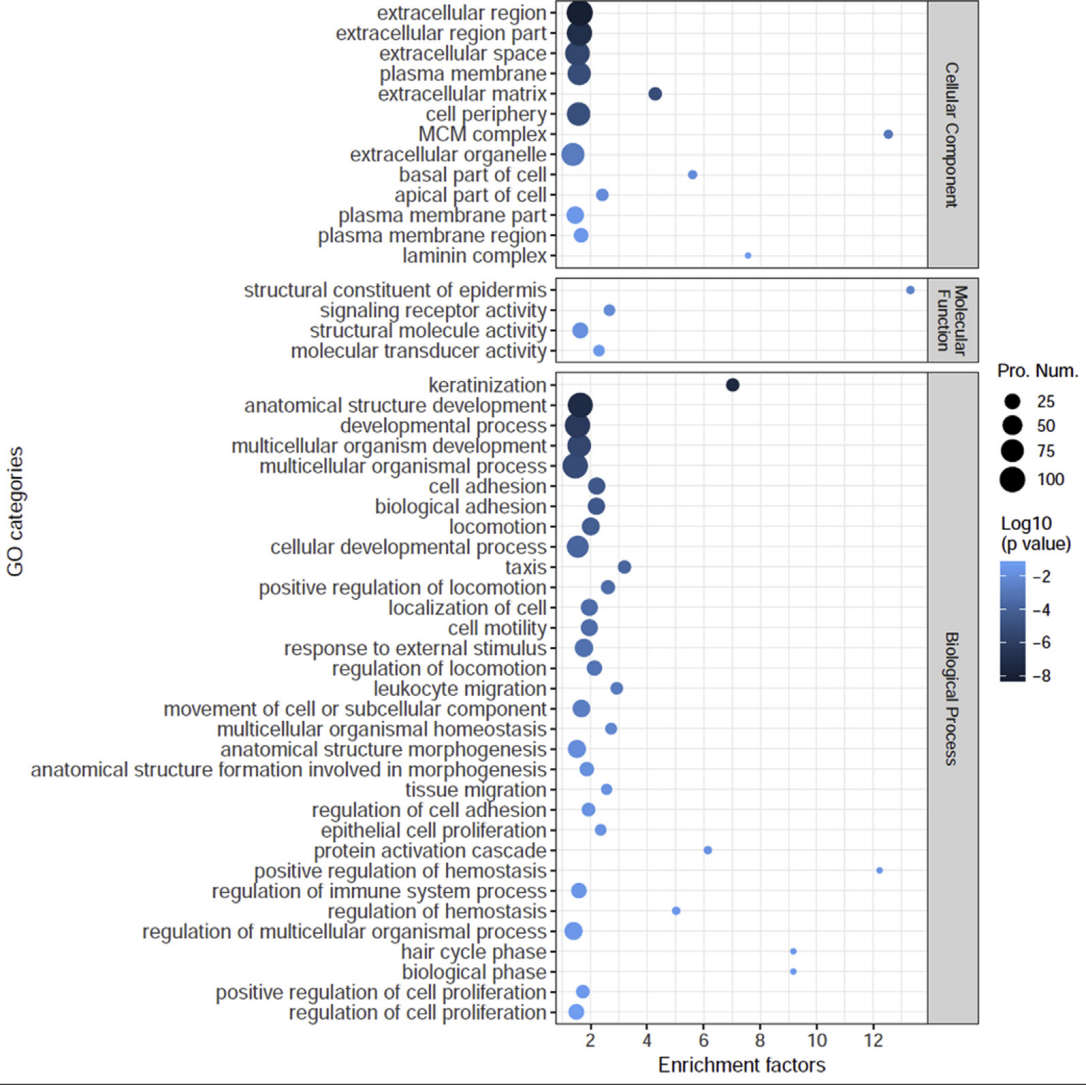
Enriched GO categories of up/down regulated proteins in well-differentiated normal human primary bronchial/tracheal epithelial cells compared with un-differentiated cells. Classification nodes that were 1 or 2 steps from the root node are shown. The size of the dot indicates the number of proteins in the up/down regulated protein set. P values were obtained by Fisher's test.

For GO assessment, classification nodes (categories) within 5 steps of the root node were surveyed. The analysis identified 30 Cellular Component categories, e.g., extracellular region (GO:0005576), plasma membrane (GO:0005886) and apical part of cell (GO:0045177); 50 Molecular Function categories, e.g., structural constituent of epidermis (GO:0030280), signaling receptor activity (GO:0038023) and structural molecule activity (GO:0005198); and 199 Biological Process categories, e.g., cell adhesion (GO:0098609), localization of cell (GO:0051674), epithelial cell proliferation (GO:0050673) and regulation of immune system process (GO:0002682), with 94.9% (188/198), 87.4% (173/198) and 91.4% (181/198) differential protein coverage, respectively. Figure 4 shows the enrichment of GO functional entries that are relatively close to the root node.

**Figure 5:**
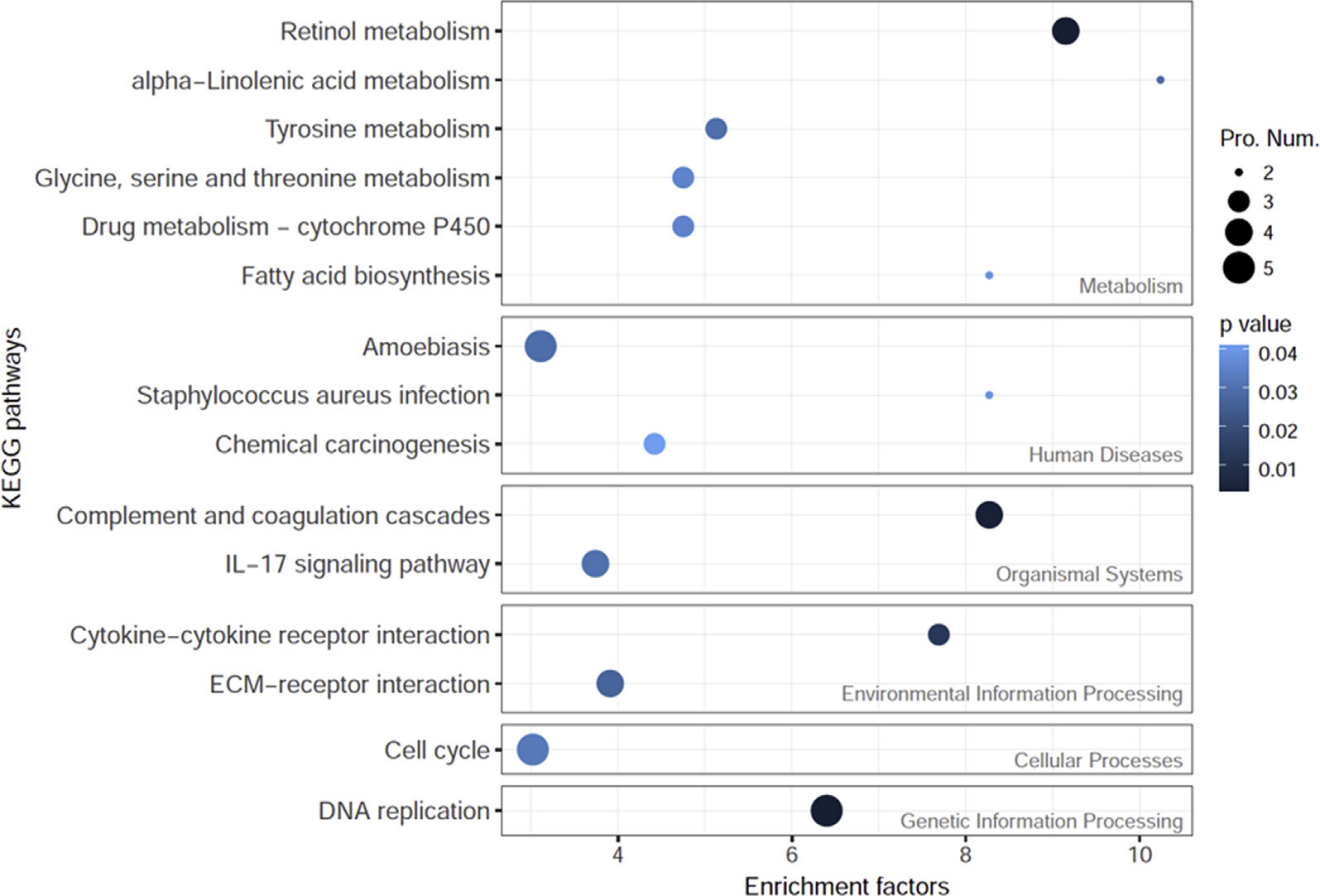
Enriched KEGG pathways of up/down regulated proteins in well-differentiated normal human primary bronchial/tracheal epithelial cells compared with un-differentiated cells. The grey labels identify the highest-order KEGG pathway classification entries. The size of the dot indicates the number of proteins in the up/down protein set. P values were obtained by Fisher's test.

Fifteen enriched KEGG pathways were detected in this study, including 6 “Metabolism” pathways, 3 “Human disease” pathways, 2 “Organismal system” pathways, 2 “Environmental information processing” pathways, one “Cellular process” pathway, and one “Genetic information processing” pathway (Figure 5).

**Figure 6:**
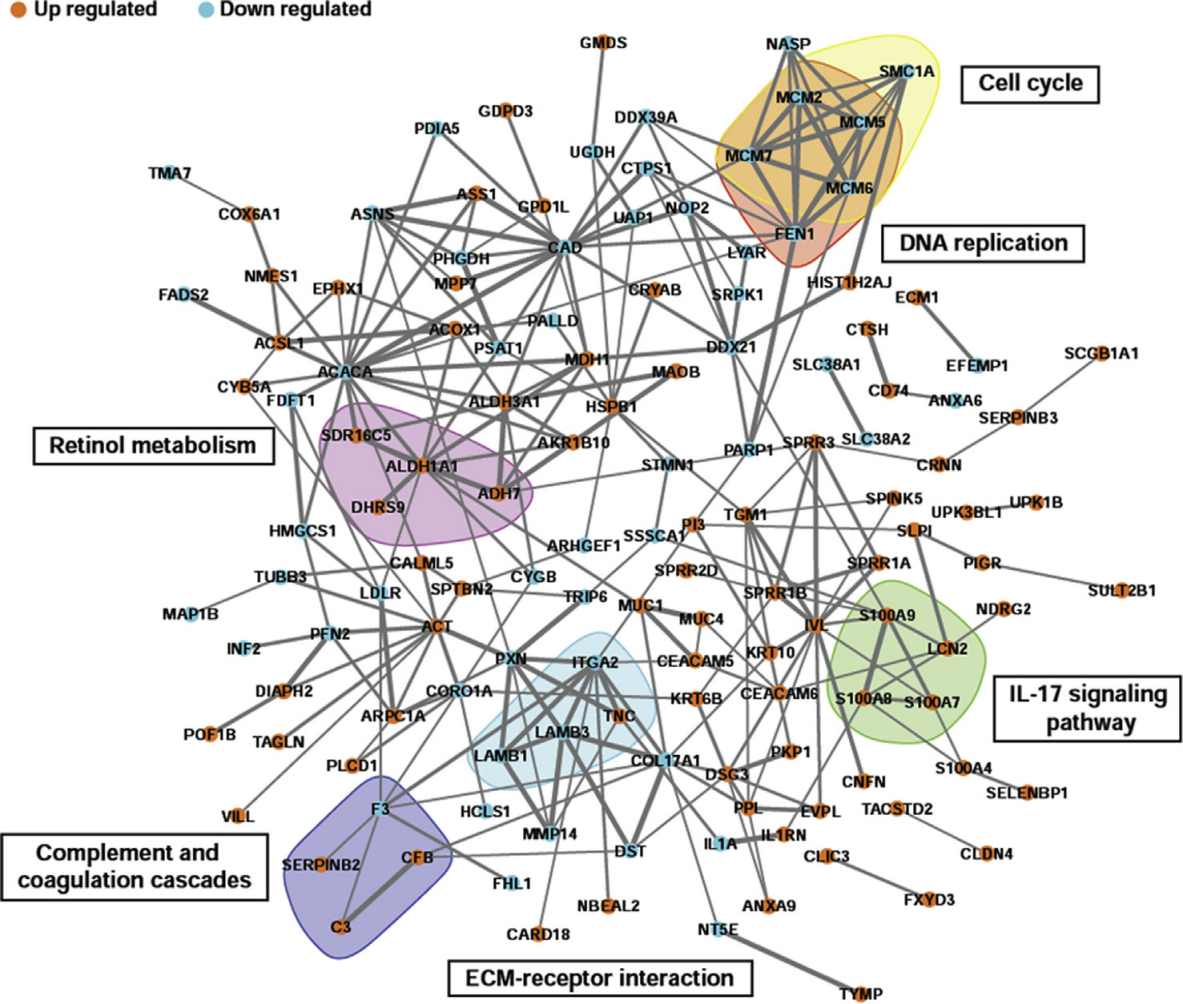
Protein-protein interaction network of up/down regulated proteins in well-differentiated normal human primary bronchial/tracheal epithelial cells compared with un-differentiated cells. Pairs of proteins with interactions are linked with lines. The thickness of the line represents the magnitude of the combined interaction score provided by STRING. Interaction scores ranged between 0.400 and 0.999. Colored blocks were assigned when a minimum of four proteins fell into the same KEGG pathway.

Query of the STRING database with the 198 up/down-regulated proteins identified 135 (68.18%) proteins with interactions and 256 pairs of interactions. These interactions constituted an integral network with several sets of divided connections (Figure 6). Six pathways of up/down-regulated proteins were identified with a minimum of four closely linked proteins in the network. Among these pathways, all proteins in the “Retinol metabolism” (ko00830) and “IL-17 signaling pathway” (ko04657) nodes were up-regulated. All proteins in the “Cell cycle” (ko04110) and “DNA replication” (ko03030) nodes were down-regulated. The “ECM-receptor interaction” (ko04512) and “Complement and coagulation cascades” (ko04610) nodes had both up- and down-regulated proteins (Figure 6).

### 3.4 Up/down-regulated protein confirmation by LC-PRM/MS

Protein expression levels of five differentially regulated proteins associated with epithelial structure formation (SCEL (O95171), KRT10 (A0A1B0GVI3) and POF1B (Q8WVV4)) (Padovano et al., 2011), cell cycle (CLIC3 (Q8IVT2)) (Qian et al., 1999), and immunity (S100A8 (P05109)) (Ryckman et al., 2003) were verified by label-free LC-PRM/MS analysis. Experiments were performed with 12 peptides of the 5 target proteins in WD and UD cells. The relative levels of target proteins were calculated based on the corresponding peptides (Table 3). Consistent with previous proteomics results, four up-regulated proteins (SCEL, S100A8, KRT10, POF1B) and one down-regulated protein (CLIC3) were identified in WD cells compared with UD cells.

**Table 3:** LC-PRM/MS confirmation of up/down-regulated proteins in well-differentiated normal human primary bronchial/tracheal epithelial cells compared with un-differentiated cells.

UniProtKB Accession	Protein name	Protein description	Mean content in WD cells	Mean content in UD cells	Content ratio (WD/UD)	P value
O95171	SCEL	Sciellin	6.0974	0.1069	57.0371	0.0334
P05109	S100A8	Protein S100-A8	296.1361	4.6353	63.8869	0.0167
A0A1B0GVI3	KRT10	Keratin, type I cytoskeletal 10	0.1159	0.0092	12.6386	0.0089
Q8WVV4	POF1B	Protein POF1B	1.6924	0.1248	13.5604	0.0027
Q8IVT2	CLIC3	Mitotic interactor and substrate of PLK1	0.3517	1.4983	0.2347	0.0005

WD and UD cells: well-differentiated and un-differentiated normal human primary bronchial/tracheal epithelial cells.

Supporting data from this study are available in supplementary materials (Tables S1 and S2).

## 4 Discussion

Human airway epithelium is a primary barrier to environmental exposures and signals to other cell types within the context of the epithelial mesenchymal trophic unit (Hiemstra et al., 2018). This layer plays a key role in airway remodeling and inflammation. WD cells are an important *in vitro* model for human airway epithelium which have been used in gene therapy studies, host defense studies, gene expression analysis, preclinical drug development, airborne toxicant studies and bio-defense model development. WD cells can be derived by culturing UD cells at an ALI (Ghio et al., 2013). These *in vitro* derived WD cells exhibit polarized epithelium with good barrier function (transepithelial resistance), secretory phenotype (mucin secretion) and ciliogenesis, much like epithelial cells *in vivo* (Hiemstra et al., 2018; Jiang et al., 2018). The differentiation of UD cells to WD cells involves down and up regulation of multiple genes and changes in cellular protein composition. To understand the protein profile of WD cells, we performed label-free LC-MS/MS analysis comparing protein patterns of UD and WD cells.

In this study, 33 transwell inserts of WD cells were divided into 3 samples for LC-MS/MS analysis. The mixture of WD cells in one sample was used to reduce error between experimental samples. We confirmed that cells were well-differentiated by testing TEER value (>1,000 Ω cm^2^) and expression of the biomarkers ZO-1 and β-tubulin IV (Figure 1). Proteins differentially expressed in WD cells compared with UD cells were identified by label-free LC-MS/MS and confirmed by LC-PRM/MS (Table 3).

A total of 3,579 proteins were identified in the six samples. Principal components of WD and UD cells exhibited considerable separation (Figure 2), suggesting substantial difference between the two cell types. Our analyses identified 198 proteins that were significantly different between the two cell types (Figure 3), including 121 up-regulated and 77 down-regulated proteins in WD cells (Table 1, Table 2). GO analysis of the differentially expressed proteins classified the proteins into structure formation of epithelium, cell cycle and immunity (Figure 4). Membrane-associated proteins were heterogeneous, including plasma membrane (GO: 0005886), and extracellular region (GO: 0005576) proteins (Figure 4, Table 1, Table 2) with a myriad of functions, e.g. structure formation (e.g. SPRR1B (P22528), SPRR2D (P22532)) (Steinert and Marekov, 1995), signal transduction (e.g. CD74 (P04233)) (Leng et al., 2003), substance transport (e.g. GPD1L (Q8N335)) (Valdivia et al., 2009), and immune recognition (e.g. HLA-DRB1 (Q5Y7A7)) (Ooi et al., 2017). These differentially expressed proteins could be of great significance in understanding the physiological functions of airway epithelium. In addition, the results of the current study provides important candidate proteins that may be associated with selective infection of WD cells versus UD cells, e.g. human bocavirus (Qiu et al., 2017).

Six of the 15 enriched KEGG pathways, “Retinol metabolism”, “IL-17 signaling pathway”, “Complement and coagulation cascades”, “ECM-receptor interaction”, “Cell cycle”, and “DNA replication” had a minimum of four closely linked differentially expressed proteins (Figures 5 and 6). Of these, the highest EF was observed in the down-regulated minichromosome maintenance (MCM) proteins (GO: 0042555) MCM7, MCM5, MCM2 and MCM6 (Table 2, Figure 4). These proteins have been reported to contain an ATPase motif (Davey et al., 2003), and are important in DNA replication and cell cycle (Figure 6). These proteins may therefore contribute to the low proliferation levels of WD cells (Jiang et al., 2018; Min et al., 2016).

Four proteins (ADH7, DHRS9, SDR16C5, ALDH1A1) in the retinol metabolism pathway were up-regulated in WD cells (Table 1, Figure 6). Up-regulation of retinol dehydrogenase activity could enhance retinoic acid production (Liden and Eriksson, 2006). Retinoic acid regulates a variety of genes, plays important roles in cell growth, differentiation, and organogenesis (Balmer and Blomhoff, 2002; Blomhoff et al., 1991), and is important for mucosal immunity regulation (Penkert et al., 2017; Sirisinha, 2015).

Four proteins (S100A7, S100A8, S100A9, LCN2) in the IL-17 signaling pathway were also up-regulated in WD cells (Table 1, Figure 6). S100A7, S100A8 and S100A9 are calcium- and zinc-binding proteins which play a prominent role in the regulation of inflammatory processes and immune response. These proteins can induce neutrophil chemotaxis and adhesion (Miyasaki et al., 1993; Ryckman et al., 2003). LCN2 is an iron-trafficking protein involved in multiple processes, e.g. apoptosis, innate immunity and renal development (Bao et al., 2010; Shields-Cutler et al., 2016; Yang et al., 2002). Up-regulation of the four IL-17 pathway proteins could increase antimicrobial activity of WD cells.

The other two KEGG pathways with four or more closely linked differential proteins were ECM-receptor interaction and complement and coagulation cascades (Figures 5 and 6). The presence of up- and down-regulated proteins in both these pathways indicates that WD cells have significantly different cell junction, extracellular matrix composition, and immune response compared with UD cells (Martina et al., 2010; Na et al., 2016; Slade et al., 2013).

The current study does have some limitations. First, human respiratory epithelium is complex exhibiting large variation in different regions of the tissue. The current study evaluated bronchial/tracheal epithelial cells. Second, cells from one donor were used for all evaluations in the current study. Cells from different individuals may have the potential to change results. Despite these considerations, this study provides a global proteomic profile of WD and UD cells. These results provide insights about differential protein profiles in un-differentiated and well-differentiated bronchial/tracheal epithelial cells and can help future studies.

## 5 Conclusions

WD cells are an important *in vitro* human airway epithelial model that can be derived by culturing UD cells at an air-liquid interface. In this work, we analyzed the proteomic profiles of WD and UD cells. A total of 3,579 proteins were identified in WD and UD cells. Of these, 198 proteins were found to be differentially expressed, with 121 proteins up-regulated and 77 proteins down-regulated in WD cells compared with UD cells. Most of the differentially expressed proteins were enriched in categories related to structure formation of epithelium, cell cycle, and immunity. This study provides the protein profiles of WD and UD cells increasing knowledge of proteins associated with human airway epithelium.

## Declarations

### Author contribution statement

Wen-Kuan Liu: Conceived and designed the experiments; Performed the experiments; Analyzed and interpreted the data; Wrote the paper.

Duo Xu, Yun Xu, Shu-Yan Qiu, Li Zhang: Performed the experiments.

Hong-Kai Wu: Conceived and designed the experiments; Analyzed and interpreted the data; Wrote the paper.

Rong Zhou: Conceived and designed the experiments; Wrote the paper.

### Funding statement

This work was supported by the 10.13039/501100001809National Natural Science Foundation of China [grant numbers 81970003, 31500143]; the National Science and Technology Major Project of China [grant numbers 2018ZX10102001, 2017ZX10103011]; and 10.13039/501100004000Guangzhou Science and Technology Program key projects [grant number 201803040004].

### Competing interest statement

The authors declare no conflict of interest.

### Additional information

No additional information is available for this paper.

## Appendix A Supplementary data

Table_S1 Differential proteins enriched GO categories in detail. Table_S1

Table_S2 Differential proteins enriched KEGG pathways in detail. Table_S2
